# Towards sub-quadratic time and space complexity solutions for the dated tree reconciliation problem

**DOI:** 10.1186/s13015-016-0077-5

**Published:** 2016-05-21

**Authors:** Benjamin Drinkwater, Michael A. Charleston

**Affiliations:** School of Information Technologies, University of Sydney, 1 Cleveland St, Sydney, 2006 NSW Australia; School of Physical Sciences, University Of Tasmania, Hobart, 7005 Tasmania Australia

**Keywords:** Coevolution, Phylogny, Cophylogeny, Tree reconciliation, Tree shape, NP-hard

## Abstract

**Background:**

Recent coevolutionary analysis has considered tree topology as a means to reduce the asymptotic complexity associated with inferring the complex coevolutionary interrelationships that arise between phylogenetic trees. Targeted algorithmic design for specific tree topologies has to date been highly successful, with one recent formulation providing a logarithmic space complexity reduction for the dated tree reconciliation problem.

**Methods:**

In this work we build on this prior analysis providing a further asymptotic space reduction, by providing a new formulation for the dynamic programming table used by a number of popular coevolutionary analysis techniques. This model gives rise to a sub quadratic running time solution for the dated tree reconciliation problem for selected tree topologies, and is shown to be, in practice, the fastest method for solving the dated tree reconciliation problem for expected evolutionary trees. This result is achieved through the analysis of not only the topology of the trees considered for coevolutionary analysis, but also the underlying structure of the dynamic programming algorithms that are traditionally applied to such analysis.

**Conclusion:**

The newly inferred theoretical complexity bounds introduced herein are then validated using a combination of synthetic and biological data sets, where the proposed model is shown to provide an $O(\sqrt{n})$ space saving, while it is observed to run in half the time compared to the fastest known algorithm for solving the dated tree reconciliation problem. What is even more significant is that the algorithm derived herein is able to guarantee the optimality of its inferred solution, something that algorithms of comparable speed have to date been unable to achieve.

## Background

Selective pressures and the adaptations that they give rise to, have provided almost a limitless diversity within the natural world [1]. These adaptations are often represented using bifurcating trees, where each internal node represents a divergence of a species lineage. Modelling of the evolutionary process in this manner, in particular the rates and patterns with which species diverge, is encapsulated within the field of phylogenetics [2]. A long standing area of interest in this field is the unbalanced nature of evolutionary trees, and the relationship between this imbalance and the evolutionary process [3]. Analysis of this imbalance can be traced back to at least Yule’s modelling of the evolutionary process in 1924 [4], yet even today, more than ninety years after Yule’s model was first proposed, there still remains no single synthetic model capable of successfully capturing the topological variation of the evolutionary process [5], although significant progress has been made recently including the introduction of the the age dependent model by Hagen et al.[6]. While no single model is able to capture the variation of all evolutionary trees, it is possible to bound this variation using the *Yule* and *Uniform* synthetic tree generation models [7, 8]. As such, targeted algorithmic development has been able to exploit this narrow subset of expected topologies as a means to optimise phylogenetic analysis techniques for expected evolutionary data [9].

The Yule model, also known as the equal-rates-Markov model [7, 10], is a synthetic tree generation process, which produces trees through a continuous-time pure birth process where each node has the same instantaneous rate of speciation, regardless of the length of time since its parent speciated. Ignoring branch lengths when selecting the next node for speciation has been shown to produce trees that represent the most balanced evolutionary trees within the tree of life [11, 12].

The Uniform model, also known as the proportional-to-distinguishable arrangements (PDA) model [13], is a synthetic tree generation process that produces trees through uniform sampling of all possible tree shapes [8]. Although the Uniform model captures the behaviour of a number of biological processes, such as explosive radiation [14] and multitype branching processes with species quasi stabilization [15], it does not directly model any evolutionary process, nor does it, in its purest sense grow trees [5]. While this model may not simulate the evolutionary process directly, it does provide a bound for the most unbalanced phylogenetic trees [16, 17].

Only recently has tree topology, specifically the Yule and Uniform models, been considered as a means to reduce the computational complexity associated with the analysis of coevolving systems. Tree topology, however, may be leveraged for such analysis, as coevolution considers the relationships between two or more phylogenetic trees. One popular coevolutionary analysis approach where tree topology may be exploited is *cophylogeny mapping*, due to the high correlation between this technique’s computational complexity and the shape of phylogenetic trees [9].

Cophylogeny mapping is the process of mapping a dependent (parasite) phylogeny into an independent (host) phylogeny, providing a framework to analyse the significance of the observed congruence between a pair of phylogenetic trees, and reconcile the shared evolutionary history for the two phylogenetic trees in question. This reconciliation process generally applies four recoverable evolutionary events: *codivergence*, *duplication*, *host switch* and *loss* [18].

These four evolutionary events allow for the shared evolutionary history to be inferred, regardless of any form of incongruence that may exist between the pair of evolutionary trees [19]. In fact Ronquist in 1995 [18] proved that these four evolutionary events alone are sufficient to reconcile all conceivable phylogenetic tree pairings if the problem instance is constrained, such that a parasite may only inhabit a single host; the version of the problem considered herein, and throughout the majority of coevolutionary analysis literature to date [20–37].

The development of algorithms which map a dependent phylogeny into an independent phylogeny has gained significant traction due to its extensibility for a number of important problems in the field of evolutionary biology, including gene–species tree reconciliation, where the evolutionary events considered within this context are cospeciation, gene duplication, lateral gene transfer and loss [38–42], and biogeographical reconciliation, where the evolutionary events considered within this context include allopatric speciation, sympatric speciation, dispersal, and extinction [43–47].

Cophylogeny mapping algorithms are developed with the purpose of inferring a minimum cost map, where each evolutionary event is assigned an associated penalty score, where the minimum cost map aims to represent the most likely shared coevolutionary history between a pair of phylogenetic trees [48]. The minimum cost may be defined as follows [21]:

1 $$\begin{aligned} E = \alpha C + \beta D + \gamma W + \delta L \end{aligned}$$

where *C*, *D*, *W*, and *L* define the associated penalty cost for codivergence, duplication, host switch and loss events respectively, and $\alpha$, $\beta$, $\gamma$ and $\delta$ define the number of codivergence, duplication, host switch and loss events within the reconciled map [49].

**Fig. 1:**
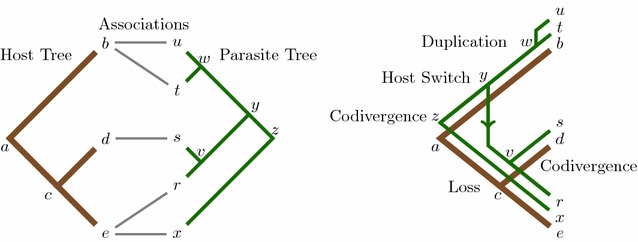
Tanglegram instance and one of its optimal maps. The resultant map (*right*) of a pair of phylogenetic trees based on their known associations (*left*). What is special about this particular map is that it is composed of all four evolutionary events, codivergence (at nodes *z* and *v*), duplication (at node w), host switch (at node *y*) and loss (edge (*z*, *x*) at host node *c*)

An example of a map that displays all four evolutionary events can be seen in Fig.  1. This map has been inferred from an input set, known as a tanglegram, which consists of a host tree, parasite tree and the associations between their extant taxa [26]. In this example, the inferred map is optimal under a certain set of cost schemes, most notably where the event costs are set under the *Jungle* cost scheme [25, 50], one of the most commonly applied cost schemes in coevolutionary analysis [26, 51–54].

The inference of a map which minimises *E*, often referred to as the cophylogeny reconstruction problem [55], is NP-Hard [56]. Due to the inherent computational complexity associated with this problem, coevolutionary analysis has often been forced to rely on heuristics [27, 29–36, 55]. There are currently two popular heuristics applied to solve the cophylogeny reconstruction problem where the first ignores the order of evolutionary events defined by the parasite phylogeny [27], and the second constrains the order of evolutionary events within the host phylogeny, reducing this problem to the polynomially solvable *dated tree reconciliation problem* [55]. It is the latter that is considered in detail herein.

A major advantage of fixing the internal node ordering is that it provides a framework that may guarantee that the reported solutions are biologically feasible, something that alternate methods have been unable to achieve [32, 33]. Constraining the problem in this fashion however presents its own challenges, most notably that there are an exponential number of internal node orderings in the worst case that needs to be considered to guarantee optimality [55]. Metaheuristics, however, have recently been shown to provide a successful means to mitigate this complexity, where genetic algorithms in particular, have been shown to converge on robust estimations in a reasonable period of time [29]. Due to the success of genetic algorithms for solving the cophylogeny reconstruction problem, recent algorithmic analysis has focused on minimising the associated time complexity of the dated tree reconciliation problem to allow for a greater exploration of this complex search space within a fixed period of time [36, 48].

To date the fastest known approach for solving the dated tree reconciliation problem is Bansal et al.’s reconciliation algorithm, implemented in the software suite RANGER-DTL [57]. This $O(n^2\log {n})$ algorithm requires $O(n^2)$ space, offering a significant reduction from the initial solution proposed by Libeskind-Hadas and Charleston in 2009 ($O(n^7)$) [55]. It is important to note, however, that Bansal et al.’s reconciliation algorithm achieves this complexity bound by relaxing the constraint that the reconciled map is time-consistent [34]. To guarantee the consistency of the inferred map requires a cubic time algorithm as defined by Yodpinyanee et al.’s Edge Mapping algorithm [31], Doyon et al.’s Slicing algorithm [32, 33] or Drinkwater and Charleston’s Improved Node Mapping algorithm [49].

Although a number of improvements have been applied since Libeskind-Hadas and Charleston’s proposal of applying the dated tree reconciliation for coevolutionary analysis [55], allowing for even larger data sets to be analysed [58], further reductions to the time and space complexity bound for the dated tree reconciliation problem would allow a greater exploration of the complex coevolutionary analysis landscape. In particular, reducing the space requirements would allow for a larger number of instances to be considered in parallel, while reducing the time complexity allows for a greater number of iterations to be performed in the same period of time.

The work presented herein re-examines the recent complexity analysis, which aims to exploit the subset of tree topologies which represent expected evolutionary trees, as a means to reduce the asymptotic complexity of the dated tree reconciliation problem [9]. Using a number of previous results we construct a new worst case complexity bound which outperforms Bansal et al.’s reconciliation [34] method for a select set of tree topologies, where these selected tree topologies represent the bounds for expected evolutionary data. As a result, the formulation presented herein represents the most efficient approach for solving the dated tree reconciliation problem, in terms of both time and space, for expected biological data. The asymptotic bounds constructed within the methodology section are then evaluated using a combination of synthetic and biological data, validating that the proposed algorithm is not only more efficient theoretically, but is also superior in practice compared to Bansal et al.’s reconciliation algorithm [34], which until now was the best known approach for solving the dated tree reconciliation problem.

## Methods

### Considering only a fraction of all bifurcating trees

When considering all possible bifurcating trees one is presented with a diverse range of tree topologies [59]. Within the field of phylogenetics only a small subset of these topologies are of interest, that is, the tree topologies which represent biological data. Trees produced under the Yule and Uniform models represent topological bounds for this subset, representing the search space of interest within the context of phylogenetics, and therefore represent the topological bounds considered herein.

**Fig. 2:**
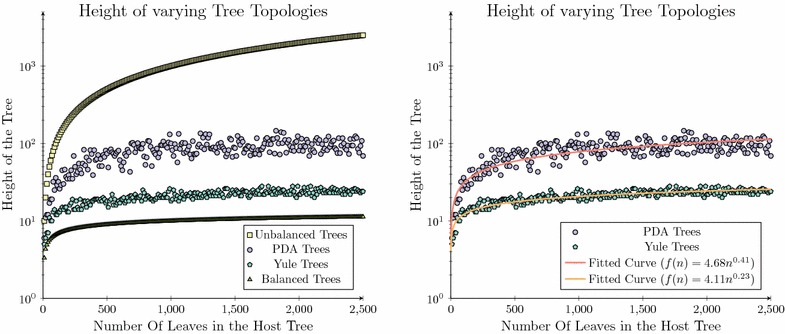
The heights of trees produced under a Yule and Uniform model. The heights of trees produced under a Yule and Uniform model bounded between the maximum and minimum heights of all possible binary trees (*left*) and the heights of trees produced under a Yule and Uniform model compared against the fitted *curves* for this specific data set inferred using least-squares function approximation (*right*)

This subset of trees represents only a fraction of all conceivable trees, highlighted in Fig.  2 (left), where the heights of a series of synthetically generated trees produced under the Yule and Uniform models are plotted between the two boundaries formed by the expected height of the most balanced and unbalanced binary trees ($\log {n}$ and $n-1$). This is compared to the trees produced under the Yule or Uniform models which are bound by the curves $4.11n^{0.23}$ and $4.68n^{0.41}$ as can be seen in Fig.  2 (right).

Using these approximate bounds we can estimate the fraction of the search space that is of interest within the context considered herein, $S_*(n)$, and compare this to the entire tree space, $S_T(n)$. When constraining the integral to only compare trees produced between 10 and 2500 taxa, for which we have data, the region of interest is only 4.99 % ($\frac{154632}{3097866}$) of the possible tree heights.

2 $$\begin{aligned} S_*(n)&= 4.68\int _{10}^{2500} (n^{0.41}) dn - 4.11\int _{10}^{2500} (n^{0.23}) dn \nonumber \\&\approx 154632 \end{aligned}$$

3 $$\begin{aligned} S_t(n)&= \int _{10}^{2500} (n-1) dn - \int _{10}^{2500} (\lg {n}) dn \nonumber \\&\approx 3097866 \end{aligned}$$

We argue that although this is a very crude estimation, it does demonstrate that the set of trees corresponding to the heights that we expect to see under realistic biological conditions represents only a very small fraction of all tree topologies. This may be extended further, where it is observed that this fraction of tree topologies only decreases in size relative to the search space encapsulating all conceivable trees, as the size of the trees considered continues to grow. This is proven by evaluating Eq.  (4) using L’Hôpital’s rule.

4 $$\begin{aligned} \lim _{x\rightarrow \infty } \frac{S_*(n)}{S_t(n)}&= \lim _{x\rightarrow \infty } \frac{\int _{10}^{x} (4.68n^{0.41}- 4.11n^{0.23}) dn}{\int _{10}^{x} (n - \lg 2{n} - 1) dn} \nonumber \\&= 0 \end{aligned}$$

This demonstrates that expected evolutionary data represents a relatively small fraction of the entire tree space and that algorithmic design may potentially benefit from leveraging its unique structure, rather than designing algorithms for the general case. This topic is explored in detail herein where we will show that this benefit may be realised through the careful construction of the dynamic programming table when reconciling a pair of phylogenetic trees which lie within the interval bounded by the Yule and Uniform models.

### Leveraging the tree topologies of the Yule and Uniform models

Leveraging tree topology as a means to mitigate the high computational complexity faced when reconciling a pair of phylogenetic trees was first proposed by Drinkwater and Charleston [49] when they introduced a logarithmic space complexity reduction for the improved Node Mapping algorithm. The space complexity reduction was achieved by applying an array of lists, rather than a two dimensional matrix to store the dynamic programming table when solving the dated tree reconciliation problem. This approach was able to exploit the previously observed distribution of elements stored within the dynamic programming tables produced using the dated tree reconciliation problem; in particular that significantly fewer than the $O(n^2)$ possible elements are stored using this technique [49].

**Fig. 3:**
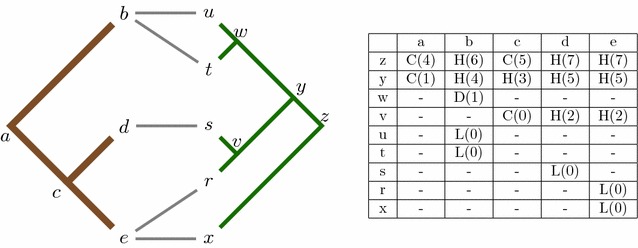
Tanglegram instance and the matrix which corresponds to the optimal map presented in Fig. 1. The tanglegram (*left*) first presented in Fig.  1 along with the dynamic programming matrix (*right*) generated in the process of solving this tanglegram instance using the Improved Node Mapping algorithm. What is of interest is that only 19 of the possible 45 (42 %) elements in the matrix are populated to solve this particular example. It is this gap, number of unpopulated elements within the dynamic programming matrix, which is exploited within this analysis

Such a distribution can be observed in Fig.  3, where the dynamic programming table required to solve the tanglegram instance introduced in Fig.  1 has been constructed. In this case fewer than half of the total number of elements are populated (only 42 %), where it has been shown that this observed distribution is asymptotically less than $O(n^2)$ [9]. To infer this asymptotic bound, Drinkwater and Charleston [9] proposed an arithmetic series which considers the aggregate of two functions *f*(*i*) and *g*(*i*). The function *f*(*i*) captures the number of elements, *mapping sites*, retained within the dynamic programming table for each parasite node, at level *i*; where the *level* is the maximum distance that that parasite node is from its leaves. The function *g*(*i*) captures the number of nodes at a specific level *i* within a bifurcating tree. This series therefore summarises the total number of elements retained when solving the dated tree reconciliation problem; where this series may be represented in the form:

5 $$\begin{aligned} \sum \limits _{i=0}^{h}(f(i) \times g(i)) \end{aligned}$$

where *h* is the height of the parasite tree; bound between $\log {n}$ and $n-1$.

It is known that *g*(*i*) varies depending on the tree topology considered, ranging from $g(i) = 1$ for completely unbalanced trees through to $g(i) = n \times 2^{-i}$ for completely balanced trees. It is this variation that may be leveraged when designing coevolutionary analysis algorithms, where they may be targeted exclusively for expected evolutionary data. This may be achieved by applying the known formulations for *g*(*i*) for trees produced under expected Yule and Uniform models which have been previously derived as:

#### **Theorem 1**

*The expected number of internal nodes at each level of a tree generated under a Yule process is *$\frac{2^{(i-1)}n}{3^i}$*, wherenis the number of leaves in the tree andi is the level for all*$i \ge 1$*, (Drinkwater and Charlteston * [9]).

#### **Theorem 2**

*The expected number of internal nodes at each level of a tree generated under a Uniform process is *$\frac{3^{(i-1)}n}{4^i}$*, wheren is the number of leaves in the tree and i is the level for all *$i \ge 1$*, (Drinkwater and Charlteston * [9]).

By inferring targeted formulations for *g*(*i*), which leverage topological properties of expected evolutionary data, it has been shown that a sub-quadratic number of elements are retained when solving the dated tree reconciliation problem. Within this work we build on the prior formulations using Eq. (5), where, rather than achieving a logarithmic space saving, we introduce a square root space complexity reduction. This is achieved by observing that while prior asymptotic analyses have inferred a tight bound for *g*(*i*), a rather loose set of constraints was applied in the formulation of *f*(*i*), specifically *f*(*i*) was defined under Libeskind-Hadas and Charleston’s 2009 [55] formulation of the Node Mapping algorithm, rather than the Improved Node Mapping algorithm [49] introduced in 2014.

### Carefully constructing the dynamic programming table

Node mapping algorithms construct dynamic programming tables by mapping each parasite node *p* into the host tree, from the leaves up to the root. This is contrasted with Bansal et al.’s reconciliation algorithm, which maps each parasite into the host phylogeny starting at the root, moving down to the tips, resulting in a significant reduction in the algorithm’s time complexity. This is possible due to the application of a novel $O(n\log {n})$ preprocessing step, executed for each parasite node. While asymptotically slower, bottom-up, tips to root, approaches are capable of solving the dated tree reconciliation problem in sub-quadratic space, a result which has yet to be replicated for top-down, root to tips, approaches, due to their quadratic space requirement for preprocessing [9].

Previous approaches[9] which solve the dated tree reconciliation problem using sub-quadratic space, have constructed their asymptotic space complexity bound by considering the number of mapping sites required at each level in the reconciliation process, where it has been proven that the maximum number of mapping sites that must be retained for a specific node *p* at level *i* may be defined as follows:

6 $$\begin{aligned} f(i) = \left\{ \begin{array}{l l} 1 &{} \quad \text{ if } i = 0\\ \min (3^{(2^{i}-1)}, n) &{} \quad \text{ if } i \ge 1\\ \end{array}\right. \end{aligned}$$

We will show that while this exponential function does provide a bound for the number of mapping sites required for each node *p* at level *i*, it often significantly over–counts this value. The set of filters introduced herein aims to combat the rate of growth of *f*(*i*) to provide an asymptotic reduction to the space storage requirement for the dated tree reconciliation problem.

The function, *f*(*i*), introduced in Eq. (6), defines two possible values for the total number of mapping sites for each parasite node, *p*, at a particular level, *i*, where $i \ge 1$. Either the node *p* may be mapped to all nodes in the host tree, that is, there are *n* mapping sites required for node *p*, or there is only a subset of the host tree where *p* is mapped, where the number of nodes in this subset is bound by $3^{(2^{i}-1)} < n$. This subset can alternatively be bound by the following recurrence as defined in [9]:

7 $$\begin{aligned} a_0&= 1, \nonumber \\ a_i&= 3 \times (a_{i-1})^2 \text{ }  \forall  \text{ } i \ge 1 \end{aligned}$$

Under this model the total number of mapping sites retained are all host nodes present within the subtrees formed by the left and right children’s mapping location in the host; in line with the original construction of the Node Mapping algorithm. Under prior formulations the function, $a_i$, was constrained by noting that the number of mapping sites required is bound by *n*, but did not consider any filters to reduce the rate of growth of the recurrence relation, $a_i$.

This work considers two filters to constrain the rate at which $a_i$ grows. These filters are derived from a number of previous algorithmic optimisations applied to both the dated tree reconciliation problem and the more complex cophylogeny reconstruction problem.

The first filter considered stems from noting that only one optimal location for a codivergence or duplication needs to be retained for each parasite node *p*. This was not considered in the original construction of the node mapping algorithm [55] but has been adopted in subsequent methodologies [33, 34]. This constraint has been used not only for reducing the inherent complexity of the dated tree reconciliation problem, but also in addressing the complexity of other coevolutionary analysis techniques, starting with its application by Page in 1990 [21]. By applying this filter only one additional mapping site is considered for codivergence and duplication events when computing $a_i$ from its two children, $a_{i-1}$, in line with Page’s original formulation.

The second filter that we apply is to leverage the previous proof [49] that while there are up to $O(n^2)$ optimal host switch locations for each parasite node *p*, only one of these optimal host switch events needs to be retained to guarantee that the reconciled map is optimal. That is, through careful selection, it is possible to infer an optimal map by retaining only one host switch event[49]. This selection involves always retaining the most recent host switch event, which may be recovered using an application of the level ancestor problem [36], ensuring that the total number of loss events is minimised [49]. As noted previously this approach only guarantees that *an* optimal switch event is inferred; it cannot guarantee that *all* optimal events are inferred [49]. Therefore, when selecting a host switch event only one additional mapping location needs to be retained for $a_i$. This is the case even though a host switch may be inferred in either direction during the construction of the dynamic programming table, as at least one of those two host switch events will be mapped to the same node as its child, $a_{i-1}$, because the most recent host switch event is always selected [9]. This is consistent with a number of alternate methodologies for handling the computational intractability that may arise when dealing with host switch events, such as Bansal et al.’s [34] application of range minimum queries to infer the optimal host switch event in constant time.

It is important to note that these two filters complement one another, and that by applying both filters it can be guaranteed that an optimal reconstruction will be recovered when applied to the Node Mapping algorithm [49]. That is, that retaining only three mapping sites, a single codivergence or duplication event, along with two host switch events for each parasite node *p*, ensures that the resultant map will be optimal [33, 34, 36, 49]. The complementary nature of these two filters was proved as part of the formulation of the Improved Node Mapping algorithm, although these filters were not considered within the context of dynamically sized data structures at the time they were proposed [9, 49].

The benefit of applying these two additional filters to the formulation of $a_i$ is that it allows for a reformulation of $a_i$ as an additive growth function as opposed to the initial multiplicative function, Eq.  (7). This drastically reduces the growth of $a_i$, as follows:

8 $$\begin{aligned} a_0&= 1 \nonumber \\ a_1&= 3 \nonumber \\ a_i&= a_{i-1} + a_{i-1} + 2 \text{ } \forall \text{ } i \ge 2 \end{aligned}$$

We will show that this reformulation translates to a significant reduction to the asymptotic space complexity bound for the Improved Node Mapping algorithm. Of note, is that $a_1$ is defined as 3 rather than 4 at level one, as both host switch events will always be mapped to the host leaves in this case. This result was inferred as part of a prior formulation of $a_i$ [9].

From Eq.  (8) a new closed form function may be derived which is asymptotically less than Eq.  (6). This function allows for the total number of mapping sites required to solve the dated tree reconciliation problem to be derived as:

#### **Theorem 3**

*The maximum number of mapping sites, *$a_i$*, required to solve the dated tree reconciliation problem optimally for each level*$i \ge 1$*is bounded by the function*$a_i = 5 \times 2^{(i-1)} - 2$

#### *Proof*

9 $$\begin{aligned} a_i&= a_{i-1} + a_{i-1} + 2 \nonumber \\&= 2 \times 2 \times a_{i-2} + 4 + 2 \nonumber \\&= 2 \times 2 \times 2 \times a_{i-3} + 8 + 4 + 2 \nonumber \\&= \dots \nonumber \\&= 2^{(i-1)}a_1 + 2 \times (2^{(i-1)} - 1) \nonumber \\&= 5 \times 2^{(i-1)} - 2 \end{aligned}$$

$$\square$$

Using this result *f*(*i*) may be redefined as follows:

10 $$\begin{aligned} f(i) = \left\{ \begin{array}{l l} 1 &{} \quad \text{ if } i = 0\\ \min (5 \times 2^{(i-1)} - 2, n) &{} \quad \text{ if } i \ge 1\\ \end{array}\right. \end{aligned}$$

where this function may then be split into three components; where $i=0$, the values for *i* for which $5 \times 2^{(i-1)} - 2 < n$ and the values for *i* for which $5 \times 2^{(i-1)} - 2 \ge n$:

#### **Lemma 1**

$$5 \times 2^{(i-1)} - 2 < n$$$$\forall$$$$i < \lfloor {\lg {(n+2)}}\rfloor -1$$

#### *Proof*

11 $$\begin{aligned} 5 \times 2^{(i-1)}-2&\le n \nonumber \\ 2^{(i-1)}&\le \frac{n+2}{5} \nonumber \\ i&\le \lg {(n+2)} - \lg {5} + 1 \nonumber \\ i&\le \lfloor {\lg {(n+2)}}\rfloor - \lfloor {\lg {5}}\rfloor + 1 \nonumber \\ i&< \lfloor {\lg {(n+2)}}\rfloor -1 \end{aligned}$$

$$\square$$

which gives rise to:

12 $$\begin{aligned} f(i) = \left\{ \begin{array}{l l} 1 &{} \quad \text{ if } i = 0\\ 5 \times 2^{(i-1)} - 2 &{} \quad \text{ if } 0 < i < \lfloor {\lg {(n+2)}}\rfloor -1\\ n &{} \quad \text{ if } i \ge \lfloor {\lg {(n+2)}}\rfloor -1\\ \end{array}\right. \end{aligned}$$

**Fig. 4:**
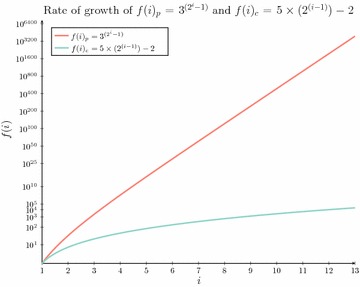
Asymptotic space comparison in practice. A comparison of two derived functions for counting the number of elements retained at each level *i*. The first $f(i)_p$ was derived as part of an early reduction to reduce the asymptotic space complexity for the dated tree reconciliation problem [9] while $f(i)_c$ is the inferred function for *f*(*i*) constructed herein and is representative of the most efficient function which has been derived for *f*(*i*) to date

This new formulation for *f*(*i*), defines a significantly larger subset of the dynamic programming table where strictly fewer than *n* sub-solutions are retained compared with previously defined functions for *f*(*i*). A visual comparison of this difference is represented in Fig.  4 where $f(i)_p$, the previous bound for *f*(*i*), is compared against $f(i)_c$, the current bound for *f*(*i*). Of note is that to accurately compare these two curves the *y* axis is configured using a double log scale.

The new formulation of *f*(*i*) derived herein clearly grows at a significantly slower rate than prior formulations. It is therefore expected that by applying our new formulation for *f*(*i*) that a further asymptotic space complexity reduction may be derived. In the next section it will be shown that this is the case with a significant reduction to not only the space required to solve the dated tree reconciliation problem but also to the time complexity as well. Most notably this new formulation is comparable in practice to the running time of Bansal et al.’s [34] reconciliation algorithm, while ensuring that reported solutions are time consistent, something which Bansal et al.’s [34] reconciliation algorithm has been unable to achieve to date.

### Complexity analysis

#### Space complexity reduction

The total storage required to solve the dated tree reconciliation problem is inferred by multiplying the total number of mapping sites retained for each node, *f*(*i*), by the total number of nodes at each level, *g*(*i*), as in Eq.  (5).

Under this model *h* represents the height of the parasite tree. To simplify this analysis the worst case height of both the Yule and Uniform trees are considered as the maximum height for all bifurcating trees, $(n-1)$, in line with prior analysis [9]. It is important to note that the heights estimated as part of the analysis undertaken in Fig.  2 would not have been appropriate as they were only representative of a small subset of tree sizes, and as such, may have poorly captured the total search space. Therefore, the complexity analysis considered herein over-counts the height of tree topologies considered, which is appropriate as this analysis only aims to provide a worst case complexity bound rather than the exact value for the number of elements retained in the dynamic programming table’s construction.

If the newly derived function for *f*(*i*) as defined in Eq.  (12) is combined with the known function for *g*(*i*) derived in [9], it is possible to expand Eq.  (5) to extrapolate the space required to solve the dated tree reconciliation problem, in the case where tanglegrams considered are composed of trees produced under a Yule (Eq.  (13)) or Uniform (Eq.  (14)) model.

13 $$\begin{aligned} O\Bigg (n + n \sum \limits _{i=1}^{\lfloor {\lg {(n+ 2)}}\rfloor -1} \frac{2^{(i-1)}(5 \times 2^{(i-1)}-2)}{3^{i}} + n^2 \sum \limits _{i=\lfloor {\lg {(n+ 2)}}\rfloor }^{n-1} \frac{2^{(i-1)}}{3^{i}}\Bigg ) \end{aligned}$$

14 $$\begin{aligned} O\Bigg (n + n \sum \limits _{i=1}^{\lfloor {\lg {(n+2)}}\rfloor -1} \frac{3^{(i-1)}(5 \times 2^{(i-1)}-2)}{4^{i}} + n^2 \sum \limits _{i=\lfloor {\lg {(n+ 2)}}\rfloor }^{n-1} \frac{3^{(i-1)}}{4^{i}}\Bigg ) \end{aligned}$$

The simplification of these two Eqs.  (13) and (14) may therefore provide a new set of worst case space complexity bounds for solving the dated tree reconciliation problem; where these simplified space complexity bounds give rise to the following two theorems:

##### **Theorem 4**

*The space required to solve the dated tree reconciliation problem for tanglegrams composed of trees constructed under the expected Yule process is bounded by*$O(n^{1.42})$.

##### **Theorem 5**

*The space required to solve the dated tree reconciliation problem for tanglegrams composed of trees constructed under the expected Uniform process is bounded by *$O(n^{1.58})$.

#### Proof for Theorem 4

##### *Proof*

To infer the worst case space complexity required to solve the dated tree reconciliation problem for coevolutionary systems, composed of phylogenetic trees produced under an expected Yule model, requires that the set of geometric series defined in Eq.  (13) be simplified. This may be achieved by treating the asymptotic complexity bound in Eq.  (13) as a function $\alpha (n)$ and simplifying as follows:

15 $$\begin{aligned} \alpha (n)&= n+n \sum \limits _{i=1}^{\lfloor {\lg {(n+2)}}\rfloor -1} \frac{2^{(i-1)}(5 \times 2^{(i-1)}-2)}{3^{i}} + n^2 \sum \limits _{i=\lfloor {\lg {(n+2)}}\rfloor }^{n-1} \frac{2^{(i-1)}}{3^{i}} \nonumber \\&= n+n \Bigg ( \frac{5}{4} \sum \limits _{i=1}^{\lfloor {\lg {(n+2)}}\rfloor -1} \bigg (\frac{4}{3}\bigg )^i - \sum \limits _{i=1}^{\lfloor {\lg {(n+2)}}\rfloor -1} \bigg (\frac{2}{3}\bigg )^i + \frac{n}{2} \sum \limits _{i=\lfloor {\lg {(n+2)}}\rfloor }^{n-1} \bigg (\frac{2}{3}\bigg )^i\Bigg ) \nonumber \\&< n+n \Bigg ( \frac{5}{4} \sum \limits _{i=1}^{\lfloor {\lg {(n+2)}}\rfloor -1} \bigg (\frac{4}{3}\bigg )^i + \frac{n}{2} \sum \limits _{i=\lfloor {\lg {(n+2)}}\rfloor }^{n-1} \bigg (\frac{2}{3}\bigg )^i\Bigg ) \nonumber \\&= n+n \Bigg ( \frac{5}{4} \times \bigg (\frac{\frac{4}{3} - \frac{4}{3}^{\lfloor {\lg {(n+2)}}\rfloor }}{1 - \frac{4}{3}}\bigg ) + \frac{n}{2} \times \bigg ( \frac{ \big (\frac{2}{3}\big )^{\lfloor {\lg {(n+2)}}\rfloor } - \big (\frac{2}{3}\big )^{n}}{1 - \frac{2}{3}} \bigg )\Bigg ) \nonumber \\&= n+n \Bigg ( \frac{15}{4} \times \bigg (\frac{4}{3}^{\lfloor {\lg {(n+2)}}\rfloor } -\frac{4}{3}\bigg ) + \frac{3n}{2} \times \bigg (\frac{2}{3}^{\lfloor {\lg {(n+2)}}\rfloor } -\bigg (\frac{2}{3}\bigg )^{n}\bigg ) \Bigg )\nonumber \\&\le n+n \Bigg ( \frac{15}{4} \times \bigg (\frac{4}{3}^{(\lg {(n+2)})} -\frac{4}{3}\bigg ) + \frac{3n}{2} \times \bigg (\frac{2}{3}^{(\lg {(n+2)})} - \bigg (\frac{2}{3}\bigg )^{n}\bigg ) \Bigg )\nonumber \\&= n+n \Bigg ( \frac{15}{4} \times \frac{4}{3}^{(\lg {(n+2)})} + \frac{3n}{2} \times \frac{2}{3}^{(\lg {(n+2)})} - n \times \bigg (\frac{2}{3}\bigg )^{n-1} -5 \Bigg )\nonumber \\&< n+n \Bigg ( \frac{15}{4} \times \frac{4}{3}^{(\lg {(n+2)})} + \frac{3n}{2} \times \frac{2}{3}^{(\lg {(n+2)})} \Bigg )\nonumber \\&= n+n \Bigg ( \frac{15}{4} \times (n+2)^{(2-\lg {3})} + \frac{3n}{2} \times (n+2)^{(1-\lg {3})} \Bigg ) \nonumber \\&\approx n \times n^{(2-\lg {3})} \nonumber \\&\approx n^{1.42} \end{aligned}$$

$$\square$$

#### Proof for Theorem 5

##### *Proof*

To infer the worst case space complexity required to solve the dated tree reconciliation problem for coevolutionary systems, composed of phylogenetic trees produced under an expected Uniform model, requires that the set of geometric series defined in Eq.  (14) be simplified. This may be achieved by treating the asymptotic complexity bound in Eq.  (14) as a function $\beta (n)$ and simplifying as follows:

16 $$\begin{aligned} \beta (n)&= n+n \sum \limits _{i=1}^{\lfloor {\lg {(n+2)}}\rfloor -1} \frac{3^{(i-1)}(5 \times 2^{(i-1)}-2)}{4^{i}} + n^2 \sum \limits _{i=\lfloor {\lg {(n+2)}}\rfloor }^{n-1} \frac{3^{(i-1)}}{4^{i}} \nonumber \\&= n+n \Bigg ( \frac{5}{6} \sum \limits _{i=1}^{\lfloor {\lg {(n+2)}}\rfloor -1} \bigg (\frac{3^i \times 2^i}{2^i \times 2^i}\bigg ) - \frac{2}{3}\sum \limits _{i=1}^{\lfloor {\lg {(n+2)}}\rfloor -1} \bigg (\frac{3}{4}\bigg )^i + \frac{n}{3} \sum \limits _{i=\lfloor {\lg {(n+2)}}\rfloor }^{n-1} \bigg (\frac{3}{4}\bigg )^i\Bigg ) \nonumber \\&< n+n \Bigg ( \frac{5}{6} \sum \limits _{i=1}^{\lfloor {\lg {(n+2)}}\rfloor -1} \bigg (\frac{3}{2}\bigg )^i + \frac{n}{3} \sum \limits _{i=\lfloor {\lg {(n+2)}}\rfloor }^{n-1} \bigg (\frac{3}{4}\bigg )^i\Bigg ) \nonumber \\&= n+n \Bigg ( \frac{5}{6} \times \bigg (\frac{\frac{3}{2} - \frac{3}{2}^{\lfloor {\lg {(n+2)}}\rfloor }}{1 - \frac{3}{2}}\bigg ) + \frac{n}{3} \times \bigg ( \frac{ \big (\frac{3}{4}\big )^{\lfloor {\lg {(n+2)}}\rfloor } - \big (\frac{3}{4}\big )^{n}}{1 - \frac{3}{4}} \bigg )\Bigg ) \nonumber \\&= n+n \Bigg ( \frac{5}{3} \times \bigg (\frac{3}{2}^{\lfloor {\lg {(n+2)}}\rfloor } -\frac{3}{2}\bigg ) + \frac{4n}{3} \times \bigg (\frac{3}{4}^{\lfloor {\lg {(n+2)}}\rfloor } -\bigg (\frac{3}{4}\bigg )^{n}\bigg ) \Bigg )\nonumber \\&\le n+n \Bigg ( \frac{5}{3} \times \bigg (\frac{3}{2}^{(\lg {(n+2)})} -\frac{3}{2}\bigg ) + \frac{4n}{3} \times \bigg (\frac{3}{4}^{(\lg {(n+2)})} - \bigg (\frac{3}{4}\bigg )^{n}\bigg ) \Bigg )\nonumber \\&= n+n \Bigg ( \frac{5}{3} \times \frac{3}{2}^{(\lg {(n+2)})} + \frac{4n}{3} \times \frac{3}{4}^{(\lg {(n+2)})} - n \times \bigg (\frac{3}{4}\bigg )^{(n-1)} -\frac{5}{2} \Bigg )\nonumber \\&< n+n \Bigg ( \frac{5}{3} \times \frac{3}{2}^{(\lg {(n+2)})} + \frac{4n}{3} \times \frac{3}{4}^{(\lg {(n+2)})} \Bigg )\nonumber \\&= n+n \Bigg ( \frac{5}{3} \times (n+2)^{(\lg {3}-1)} + \frac{4n}{3} \times (n+2)^{(\lg {3}-2)} \Bigg ) \nonumber \\&\approx n \times n^{(\lg {3}-1)} \nonumber \\&\approx n^{1.58} \end{aligned}$$

$$\square$$

#### Space saving in practice

Theorems 4 and 5 are representative of the current lowest worst case space complexity bound for an algorithm capable of solving the dated tree reconciliation problem optimally. In fact these asymptotic bounds are only the second sub-quadratic complexity bound presented for the dated tree reconciliation problem, and offer almost an $O(\sqrt{n})$ saving compared to existing techniques.

To date little focus has been given to the space required for the dated tree reconciliation problem since Yodpinyanee et al. [31] and Bansal et al. [34] independently derived quadratic space solutions. This is largely due to current data sets not being restricted by the quadratic space requirements of these two algorithms. Space however will quickly become the bottleneck in coevolutionary analysis using the dated tree reconciliation problem as data sets continue to grow. In fact, space will become the primary limiting factor, as once the threshold on a machine’s memory is exceeded, coevolutionary analysis algorithms will be unable to solve the cophylogeny reconstruction problem regardless of time allocated for the task [9].

To observe how space will become a limiting factor it is useful to consider both the impact that the current resource requirements have on the large data sets today and how this will translate to larger data sets in the future. The largest data set analysed using the dated tree reconciliation problem to date is the mutualistic coevolutionary dependence which has formed between fig trees and their pollinator wasps [58]. This data set contains approximately 200 taxa in the host tree and 300 taxa in the parasite tree.

As the dated tree reconciliation problem requires that each of the internal and external nodes are mapped into the host, a dynamic programming table of an algorithm which applies a two dimensional matrix will in this case require 239,001 ($399 \times 599$) mapping sites be retained. This is in contrast to the worst case under our proposed model which only requires 19,319 ($599 \times 399^{0.58}$) mapping sites.

In the previous calculations we took the number of nodes as $(2n-1)$ and $(2m-1)$ where *n* and *m* are the number of leaves in the host and parasite tree respectively. When comparing algorithms which require quadratic space compared to the newly proposed model a twelve fold saving is observed.

**Fig. 5:**
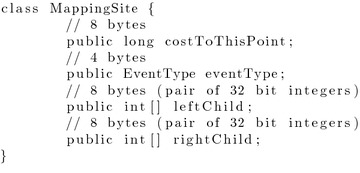
MappingSite class. The member variables of a class representing the minimum number of bytes required to store a mapping instance within the dynamic programming table

While a comparison of the number of elements in the dynamic programming table is of interest in comparing these two methodologies, what is of more interest is how this translates to the hardware requirements for the analysis of such systems. This may be estimated by multiplying the worst case number of mapping sites by the estimated size of each element stored within the dynamic programming table. Based on prior algorithmic definitions [31, 32, 49, 55] we estimated the minimal number of bytes which must be retained to reconstruct a single mapping site element which is captured in the class definition in Fig.  5.

The space of such a class is 28 bytes with the further requirement of two 32 bit words to represent the space of the class. Therefore, the total storage requirement for each *MappingSite* object is 36 bytes. When this is compared with the total number of mapping sites retained for each method the difference of each approach is 8.2 MB compared to 679 KB.

These numbers, however, only represent the space required for a single instance of the dated tree reconciliation problem. When solving the cophylogeny reconstruction problem using a metaheuristic framework it is often required that multiple instances be run in parallel. This was the case in the analysis performed over the fig-wasp system undertaken by Cruaud et al. [58] which required 1000 parallel instances.

Therefore the difference between each approach in practice is actually 8GB compared to 663 MB. Even when considering the 1000 parallel instances, a data set of this size is not impacted by its space requirement as most machines handling phylogenetic inference require more than 8GB of RAM. This difference, however, changes significantly as *n* and *m* continue to grow.

If we consider systems where the host and parasite contain 4000 taxa it becomes infeasible to apply a quadratic space solution. Coevolutionary systems of this magnitude are well within the scope of this problem as recent studies have indicated that Wolbachia has coevolved to some degree with up to 65 % of all insects [60]. As of early 2015 a small subset of this system has been constructed consisting of 397 taxa, almost twice that of the fig wasp system published three years earlier [58], and is expected to only grow further. This was demonstrated with a recent (2015) study of 3600 arthropod species and their disposition to bacterial endosymbionts, including Wolbachia [61].

In the case where metaheuristics are used to analyse systems with 4000 taxa, at least 10000 parallel instances will be required if we assume a linear scaling in the number of parallel instances which must be maintained. Such a scaling is conservative considering that a quadratic increase in the number of parallel instances was required when analysing the relationships between fig trees and their pollinator wasps, compared to the analysis of insects’ exploitation of Leguminosae, undertaken in 2010 [29]. By considering only 10,000 parallel instances the space required for each method is 20 TB compared to 492 GB respectively. While access to hardware with up to 1 TB of RAM is often feasible, having access to machines with 20 TB of RAM is far less common [62–65].

This comparison is only representative of the storage requirement for the dynamic programming table. Solutions that require quadratic space will require additional memory for preprocessing tables. These preprocessing tables will require approximately an additional 20 TB to cache the optimal evolutionary event locations. This is not the case for the algorithm described herein, which requires only linear time preprocessing [9, 49]. Therefore, sub quadratic space solutions will become critical to allow biologists to infer the underlying relationships within emerging coevolutionary systems such as Wolbachia and their arthropod hosts. Applying such algorithms offers at least a forty fold decrease in the amount of RAM required, with this only improving as the data sets considered continue to grow.

#### Time complexity reduction

Theorems 4 and 5 provide a new worst case bound for space required to solve the dated tree reconciliation problem. In this section we apply these asymptotic bounds to provide a reduction to the cubic time complexity bound faced by the Improved Node Mapping algorithm, and prove that for a select set of tree topologies it is possible to solve the dated tree reconciliation problem in sub-quadratic time.

A sub-quadratic space requirement is achieved by storing a sublinear number of mapping sites for each parasite node. A sub-cubic time complexity bound may be inferred in this same manner as the time complexity bound is also directly correlated to the number of mapping sites stored for each parasite node.

The time complexity analysis for the Improved Node Mapping algorithm considered herein is constructed in terms of the total number of host and parasite nodes, *O*(*n*). Within this context we assume the total number of nodes is approximately equal, in line with the majority of coevolutionary analysis literature [29, 55]. By treating the number of nodes in both the host and parasite trees as approximately equal the time complexity may be formulated as the total number of parasite nodes, *O*(*n*), multiplied by the total number of mapping sites, *m*, stored for each parasite node, squared [55]. The total number of mapping sites is squared as all possible pairs must be considered to ensure the optimal mapping locations are inferred [66], and therefore the time complexity bound is bound by $nm^2$.

The number of mapping sites in the worst case is *O*(*n*), however in the previous section it was shown that this value is sublinear for select tree topologies. Previous analysis considering the expected time complexity for solving the dated tree reconciliation problem has considered the distribution of *m* to be uniform [9]. If we consider the time complexity analysis in line with this prior analysis then *m*, the number of mapping sites for each parasite node, may be defined as:

##### **Lemma 2**

*The average number of mapping sites, m*,*that need to be retained for each parasite node when solving the dated tree reconciliation problem for a tanglegram composed of trees produced under a Yule model is*$n^{0.42}$.

##### **Lemma 3**

*The average number of mapping sites,m, that need to be retained for each parasite node when solving the dated tree reconciliation problem for a tanglegram composed of trees produced under a Uniform model is*$n^{0.58}$.

##### *Proof*

These results may be inferred by considering that the total space requirement $n^{1.42}$ and $n^{1.58}$ is representative of the total storage of *n* mapping sites and therefore on average there must be $n^{0.42}$ and $n^{0.58}$ stored for each mapping site respectively. $\square$

Using Lemmas (2) and (3) we may define the expected time complexity bounds for the dated tree reconciliation problem as:

##### **Corollary 1**

*The expected time required to solve the dated tree reconciliation problem for trees constructed under the expected Yule process is *$n^{1.84}$.

##### **Corollary 2**

*The expected time required to solve the dated tree reconciliation problem for trees constructed under the expected Uniform process is *$n^{2.17}$.

#### Time complexity bound in practice

The time complexity in Corollaries 1 and 2 are of interest as, for a select subset of tree topologies, those that conform to a Yule process, it is possible to solve the dated tree reconciliation problem in sub-quadratic time. In the case where trees conform to those produced by a Uniform process, however, the time complexity bound of the model presented herein is $n^{2.17}$, which is slightly worse asymptotically than Bansal et al.’s [34] $O(n^2\log {n})$ algorithm.

In practice however, the complexity of our algorithm does not exceed that of Bansal et al.’s reconciliation algorithm [34] until *n* exceeds 353 million, which is an order of magnitude greater than the current largest estimates of the number of species on our planet [67]. Further, the newly proposed algorithm can also provide the additional guarantee that all solutions reported are biologically feasible. If we compare the time complexity of the newly proposed model to those algorithms which are able to provide such a guarantee, such as Doyon et al.’s slicing model [32], this time complexity reduction is even better, offering almost an *O*(*n*) reduction in the overall time complexity.

It is important to note that the argument framed within this section is based purely on the complexity bounds of each algorithm, which may poorly represent each algorithm’s in practice performance, due to potential constants which are hidden as part of the complexity analysis performed. This discussion, however, is important as it provides further insight into the subset of tree topologies that are of interest for coevolutionary analysis, and the potential benefits that may exist when developing targeted algorithms for these data sets. In the next section we prove that the theoretical time and space complexity bounds presented herein do translate to in-practice improvements and that not only does our newly proposed algorithm out perform Bansal et al.’s [34] reconciliation algorithm in theory, but it is shown to run in less than half the time on average, using a fraction of the space.

## Results and discussion

The theoretical complexity bounds presented above prove that a significant reduction to the Improved Node Mapping algorithm’s asymptotic time and space complexity may be achieved through the careful construction of its underlying dynamic programming table. Within this section we introduce a series of datasets which aim to validate that these theoretical complexity bounds do in fact translate to improvements in practice. The validation process can be considered in three parts, where the first analysis step considers the space complexity reduction offered by the proposed model over synthetic data sets constructed using both the Yule and Uniform models. Following this the running time of the proposed method is compared against Bansal et al.’s [34] reconciliation algorithm, over the same synthetic data sets. Algorithms such as those applied within Jane [68] and MPR [69], which are the fastest methods with comparable accuracy to the proposed model, were excluded from this analysis as their cubic running times were estimated to require over 3000 computational years compared to the experimental design considered herein, which required only 24 computational hours. This is in line with prior experiments of this size [48].

In both cases the synthetic data sets applied were constructed using CoRe-PA’s random nexus file generator [30], allowing for a larger number of taxa to be considered. To allow for this study to be undertaken, CoRe-PA’s random nexus file generator was updated by the Parallel Computing and Complex Systems lab at University in Leipzig to allow for larger data sets, with the previous versions being bound to only allow up to 1000 taxa. The data set considered within this study represents one of the largest synthetic coevolutionary data sets constructed for the analysis of coevolutionary methodologies, with only the data set generated for the analysis of RASCAL offering a comparable sized data set [48].

Finally, we compare the time and space complexity of our proposed method against Bansal et al.’s [34] approach over 102 previously published biological data sets, ensuring our result translates to a time and space reduction for biological data analysis. This data set is the same as that which has been previously used to validate a number of algorithmic improvements in this field [9, 36, 48].

In all three sections a Java implementation of the Improved Node Mapping algorithm was compared against a Java implementation of Bansal et al.’s reconciliation algorithm [34]. RANGER-DTL was not used as the source code for this method is not freely available, and the aim of the analysis considered herein was to implement both algorithms using common code wherever possible, to provide the most accurate comparison of each algorithm. Implementing the experiment in this fashion results in 97 % (14259/14663 lines) of the code base being shared between both methods. Further, a benefit of using a custom implementation rather than using standalone binaries was that it allowed for the benchmarking of the specific mapping algorithms, and therefore allowed for the pre and postprocessing algorithms of each approach considered to be excluded from the time complexity analysis.

### Analysis of space complexity improvements (synthetic data)

To validate that an asymptotic space complexity reduction was achieved requires that the total number of mapping sites retained when solving the dated tree reconciliation problem be recorded over a range of tanglegrams of varying size. In this study 500 unique tanglegrams were considered including 250 tanglegram instances composed of trees produced under a Yule model, along with 250 tanglegram instances composed of trees produced under a Uniform model, where in both cases the set of tanglegrams consisted of trees ranging from 10 through to 2500 taxa. In all cases the host and parasite phylogenies were constrained, such that the size of the host and parasite phylogenies were an equivalent size.

This approach was favoured for this analysis compared to simulating coevolutionary data sets using a tool such as CoRe-Gen [70], as data sets which are randomly generated are expected to represent the entire search space more fairly [71]. Further, CoRe-PA was the most appropriate synthetic generation tool as it is the only method capable of generating synthetic coevolutionary systems using both Yule and Uniform models.

**Fig. 6:**
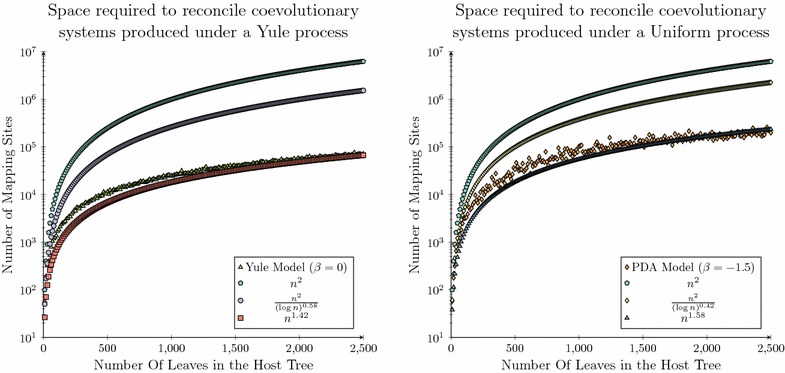
Space complexity analysis for the synthetic data set (Part 1). The space required to solve the dated tree reconciliation problem for systems composed of trees produced under a Yule (*left*) or Uniform (*right*) model

The median space required for 100 replicates of each data set has been plotted in Fig.  6, where it can be seen that significantly less than $O(n^2)$ mapping sites are required. In fact our asymptotic bounds appear to actually grow at a rate slightly faster than the required space, meaning that an even lower asymptotic complexity bound may be achievable.

As an extension to the analysis considered by Drinkwater and Charleston in 2015 [72], these results were analysed using an application of least-squares function approximation to validate that this observed phenomenon is correct [73, 74]. That is, that the observed rate of growth is even less than the theoretical space complexity bound inferred herein. In this case we restricted the least-squares analysis to only consider a power-law function, $l(n) = an^b$, fitting both *a* and *b* for both the Yule and Uniform data sets.

For this analysis a power-law function was the most appropriate function to fit to the observed data set, as *b* is expected to lie between 1 and 2. This is due to the asymptotic analysis derived herein considering a reduction from a previous quadratic bound, where this reduction may not be reduced further than a linear threshold, as each parasite node must be mapped to at least one host node. Therefore, attempting to fit a higher order polynomial function would not have been appropriate in this case.

**Fig. 7:**
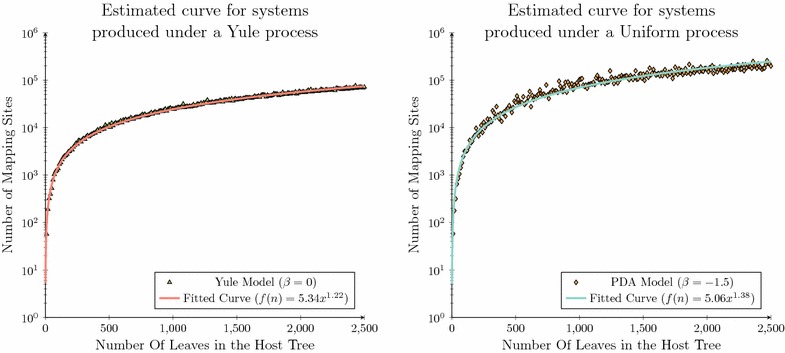
Space complexity analysis for the synthetic data set (Part 2). Fitted curves for the Yule (*left*) and Uniform (*right*) data sets using least squares analysis which have been overlaid over the observed distribution of the space required to solve the dated tree reconciliation problem

It can be seen in Fig.  7 that for both the Yule and Uniform data sets that the inferred functions provide tighter asymptotic bounds than the theoretical bounds derived herein. In the case of the Yule data set the least-squares analysis inferred the function $l(n) = 5.34x^{1.22}$, which provides a strong correlation to the observed distribution of the synthetically generated data ($R^2$ = 0.9977). A similar trend was observed for the Uniform data set. In this case the least-squares analysis inferred the function $l(n) = 5.06x^{1.38}$, which again provides a strong correlation to the observed distribution of the synthetically generated data ($R^2$ = 0.9782).

Over both the Yule and Uniform data sets least-squares analysis infers a power-law function where the parameter *b* is approximately 0.2 less than the inferred asymptotic complexity bound. This result demonstrates that there is potential for a slight improvement in the space complexity bound presented herein, while demonstrating that the space complexity analysis performed above has provided a relatively tight upper bound for the space complexity required to solve the dated tree reconciliation problem. This additional analysis also provides further confidence in the theoretical space requirements derived herein.

### Analysis of time complexity improvements (synthetic data)

**Fig. 8:**
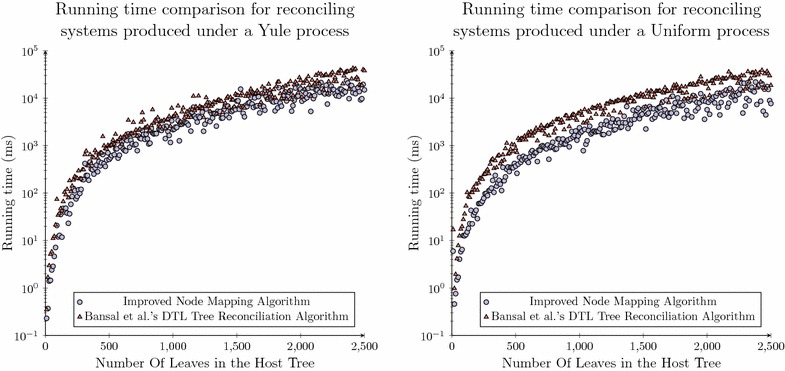
Running time analysis for the synthetic data set. A running time comparison using coevolutionary systems composed of trees produced under a Yule (*left*) or Uniform (*right*) model

The running time required to solve the dated tree reconciliation problem for each of the synthetic data sets applied in the previous section was recorded for both Improved Node Mapping and Bansal et al.’s [34] reconciliation algorithm. To ensure a robust comparison of each approach 100 replicates were run for each system with the median running time plotted in Fig.  8. The time recorded was constrained to only consider the time required to map the parasite into the host. This constraint was enforced to observe Bansal et al.’s [34] reconciliation algorithm’s running time variation for systems composed of specific tree topologies, without any potential noise that its quadratic preprocessing may introduce. This did not assist the Improved Node Mapping’s observed improvement as it applies a linear time preprocessing step compared to Bansal et al.’s, [34] quadratic preprocessing requirement, and therefore if anything this constraint provides additional benefit to Bansal et al.’s, [34] reconciliation methodology.

The running time performance of each algorithm is displayed in Fig.  8. When comparing this improvement over the entire data set it was observed that a median reduction of 43 % occurs for the Yule data set with a reduction of 62 % observed over the Uniform data set. This result is highly significant as the running time reduction is achieved using less resources, while providing a framework with a higher degree of accuracy, as the Improved Node Mapping algorithm guarantees that the solutions inferred are biologically feasible.

### Time and space complexity improvements for biological data

The time and space experiments considered for the synthetic data sets were repeated over a set of previously published biological data sets to ensure that the successful results observed over the synthetic data sets translate to biological data. The biological data considered have been applied to the validation of a number of coevolutionary analysis algorithms including TreeCollapse [36] and RASCAL [48], and includes the data sets used to validate TreeFitter [75], ParaFit [76], Tarzan [27], CoRe-PA [30], CoRe-ILP [77], and Jane [29]. In total this data set contains 102 unique tanglegrams and is the largest single source of biological data sets for coevolutionary analysis catalogued to date. These data sets are not expected to present the same degree of convergence that was observed over the synthetic data set as they are significantly smaller, with the largest only containing 53 taxa compared with 2500 in the synthetic data sets.

**Fig. 9:**
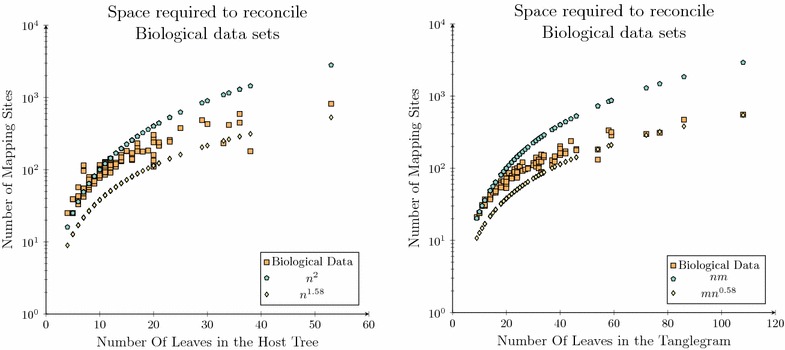
Space complexity analysis for the biological data set. The space complexity requirements for the node mapping algorithm in reference to the number of host taxa (*left*) and in reference to the total number of taxa (*right*). Note that both these plots are over the same data set

Even with the smaller biological data sets relative to the synthetic data, the experimental results demonstrate that the space required is generally less than $O(n^2)$. The exceptions seen in Fig.  9 (left) are due to larger parasite phylogenies, often twice the size of the host phylogeny, which therefore require more mapping sites as there are 2*n* sub-solutions (parasite nodes). To verify this result this experiment was rerun, where the space requirement was plotted against the size of the tanglegram (total number of taxa in both trees) rather than the number of host taxa.

This additional result provides a clearer trend that as the number of taxa within the considered data set continues to grow that there are asymptotically fewer mapping sites required compared to the quadratic number required by existing algorithms. In particular that the total number of mapping sites is asymptotically less than $n \times m$ where *n* and *m* are the number of taxa in the host and parasite phylogenies.

**Fig. 10:**
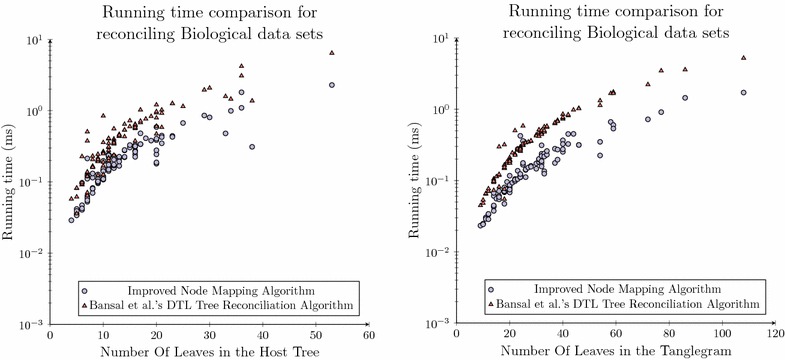
Running time analysis for the biological data set. Comparison of running times of Bansal et al.’s reconciliation algorithm and the Node Mapping algorithm over a set of previously published biological data. The *plot* on the *left* is each algorithms running time in reference to the number of host taxa in each data set while the *plot* on the *right* is each algorithms running time in reference to the total number of taxa in the tanglegram considered

In terms of running time our proposed algorithm is observed to have a median reduction of 51 % as can be seen in Fig.  10 (left). This result is a comparable reduction to that which was observed over the synthetic data set and it is expected, based on the analysis presented herein, to improve as *n* grows. The time required for both algorithms was also compared to the total size of the tanglegrams, in line with the space analysis where once again the newly proposed model was shown to outperform Bansal et al.’s reconciliation algorithm, as seen in Fig.  10 (right), this time by 57 %. These results are very promising as the newly proposed algorithm is shown to outperform Bansal et al.’s [34] reconciliation algorithm in practice, over a series of biological data sets, while providing an asymptotic space complexity reduction of $O(\sqrt{n})$.

## Conclusion

The experimental results presented demonstrate that the theoretical asymptotic reductions proved within this work translate to an in-practice time and space complexity improvement over both biological and synthetic data sets. Further, the asymptotic analysis performed herein have shown that while the Improved Node Mapping algorithm may not achieve an asymptotic reduction from its bound of $O(n^3)$ for all data sets, it is able to perform as well as Bansal et al.’s, [34], $O(n^2\log {n})$ algorithm for evolutionary data. In fact Improved Node Mapping’s running time is observed to be twice as fast as Bansal et al.’s, [34] reconciliation algorithm in practice, while providing almost an $O(\sqrt{n})$ reduction in the space required. Therefore if adopted, this approach will allow metaheuristic frameworks to execute a higher number of threads which are capable of finishing in less than half the time. Such a framework will we hope result in a higher degree of confidence in coevolutionary analysis.
